# Dietary Supplementation With the Ketogenic Diet Metabolite Beta-Hydroxybutyrate Ameliorates Post-TBI Aggression in Young-Adult Male *Drosophila*

**DOI:** 10.3389/fnins.2019.01140

**Published:** 2019-10-30

**Authors:** Derek C. Lee, Krishna Vali, Shane R. Baldwin, Jeffrey N. Divino, Justin L. Feliciano, Jesus R. Fequiere, Mirella A. Fernandez, James C. Frageau, Frank K. Longo, Salaheddine S. Madhoun, Pasquale Mingione V, Timothy R. O’Toole, Maria G. Ruiz, Geoffrey R. Tanner

**Affiliations:** ^1^Department of Physiology and Neurobiology, University of Connecticut, Storrs, CT, United States; ^2^The Connecticut Institute for the Brain and Cognitive Sciences, Storrs, CT, United States

**Keywords:** ketogenic diet (KD), traumatic brain injuries (TBI), aggression, *Drosophila*, chronic traumatic encephalopathies (CTE), KATP channel, neuroprotection

## Abstract

Traumatic brain injury (TBI), caused by repeated concussive head trauma can induce chronic traumatic encephalopathy (CTE), a neurodegenerative disease featuring behavioral symptoms ranging from cognitive deficits to elevated aggression. In a *Drosophila* model, we used a high-impact trauma device (Katzenberger et al., 2013, 2015) to induce TBI-like symptoms and to study post-TBI behavioral outcomes. Following TBI, aggression in banged male flies was significantly elevated as compared with that in unbanged flies. These increases in aggressive behavior were not the result of basal motility changes, as measured by a negative geotaxis assay. In addition, the increase in post-TBI aggression appeared to be specific to concussive trauma: neither cold exposure nor electric shock—two alternate types of trauma—significantly elevated aggressive behavior in male-male pairs. Various forms of dietary therapy, especially the high-fat, low-carbohydrate ketogenic diet (KD), have recently been explored for a wide variety of neuropathies. We thus hypothesized that putatively neuroprotective dietary interventions might be able to suppress post-traumatic elevations in aggressive behavior in animals subjected to head-trauma-inducing strikes, or “bangs”. We supplemented a normal high-carbohydrate *Drosophila* diet with the KD metabolite beta-hydroxybutyrate (β-HB)—a ketone body (KB). Banged flies raised on a KB-supplemented diet exhibited a marked reduction in aggression, whereas aggression in unbanged flies was equivalent whether dieted with KB supplements or not. Pharmacological blockade of the ATP-sensitive potassium (K_ATP_) channel abrogated KB effects reducing post-TBI aggression while pharmacological activation mimicked them, suggesting a mechanism by which KBs act in this model. KBs did not significantly extend lifespan in banged flies, but markedly extended lifespan in unbanged flies. We have thus developed a functional model for the study of post-TBI elevations of aggression. Further, we conclude that dietary interventions may be a fruitful avenue for further exploration of treatments for TBI- and CTE-related cognitive-behavioral symptoms.

## Introduction

Chronic traumatic encephalopathy (CTE) is a progressive neurodegenerative brain disorder caused by repeated traumatic brain injury (TBI), and is prevalent among contact-sport athletes (Maroon et al., 2015). Though long trivialized [cf. “punch drunk” (Martland, 1928)]—or overlooked, perhaps owing to its relatively slow onset (8–10 years) after head trauma (McKee et al., 2009)— the attendant cognitive-behavioral outcomes of CTE have attracted more recent interest following the publication of several landmark studies providing post-mortem CTE diagnosis via evidence of severe neurodegeneration in deceased former American-football players (Omalu et al., 2005, 2006; McKee et al., 2009; Mez et al., 2017). A wide variety of cognitive-behavioral disturbances manifest in cases of CTE, such as mood disorders and depression, cognitive impairment (including memory loss), and elevated aggression (McKee et al., 2012). These behaviors may be related to the high levels of neuronal death evident in cases of CTE, part of the underlying cause for which may be glutamate excitotoxicity (Blaylock and Maroon, 2011) subsequent to elevated glutamatergic neuronal signaling following brain injury (Koenig et al., 2019). We therefore hypothesized that treatments known to reduce neuronal excitability may be able to ameliorate attendant symptoms of brain injury and post-TBI progression toward CTE-like neuropathology.

The ketogenic diet (KD) is a high-fat, low-carbohydrate diet, some whose biochemical signatures (Appleton and DeVivo, 1974) mimic those of the fasted state (Foster, 1967). Mammals fed a KD exhibit elevated circulating levels of ketone bodies (KBs) such as acetoacetate and beta-hydroxybutyrate (β-HB) (Appleton and DeVivo, 1974), as well as globally elevated ATP:ADP ratios in the brain (DeVivo et al., 1978; Jarrett et al., 2008). This dietary treatment has been used intermittently for nearly 100 years (Wilder, 1921), and much more in recent decades (Thiele, 2003, 2013; Bailey et al., 2005) as an effective—albeit very challenging—therapy for drug-resistant epilepsy in children (Thiele, 2013) and, increasingly, in adults (*see*, for example, McDonald and Cervenka, 2017).

A growing body of evidence supports the idea that KBs may broadly exert neuroprotective effects (Bailey et al., 2005; Simeone et al., 2017; Elamin et al., 2018). Therefore, KD administration has gained traction in recent years as a possible therapeutic intervention for other prevalent and debilitating neurological disorders ranging from autism (Ruskin et al., 2013) to Parkinson’s Disease (Kashiwaya et al., 2000; Vanitaille et al., 2005) and Alzheimer’s Disease (AD) (Kashiwaya et al., 2013; Newport et al., 2015). Nonetheless, despite its clear efficacy in treating epilepsy, and even with substantial recent progress in understanding the KD’s mechanism of action (*see*, e.g., Masino and Rho, 2012; Lutas and Yellen, 2013; Simeone et al., 2017; Yang et al., 2019) a complete picture of the detailed molecular and cellular mechanisms underlying the presumed neuroprotective effects of KBs remains to be fully elucidated.

Nonetheless, one major promising candidate mediator of the molecular-level actions of the KD is the ATP-sensitive potassium (K_ATP_) channel (Ma et al., 2007; Tanner et al., 2011; Lutas and Yellen, 2013), whose closed state is favored by typical cellular ATP concentrations (Craig et al., 2008). A prevailing hypothesis as to how the K_ATP_ channel might function to mediate KD effects on neuropathies (especially epilepsy) is that KBs, which bypass membrane-localized glycolysis to enter directly into mitochondrial metabolism, may end up indirectly reducing ATP concentrations local to plasma-membrane K_ATP_ channels, allowing those channels to open, and thus to temper neuronal excitability via potassium efflux and the resulting membrane hyperpolarization (Yellen, 2008; Lutas and Yellen, 2013; Tantama et al., 2013).

We have developed a variation on an existing model of closed-head trauma in *Drosophila* (*see* Methods), wherein TBI is inflicted using a “high-impact trauma” (HIT) device to “bang” flies with high force (Katzenberger et al., 2013, 2015). Following high-impact concussion, banged flies exhibit readily observable symptoms similar to those presenting in human TBI patients (Martland, 1928; Pellman, 2003; Pellman et al., 2004): temporary incapacitation and ataxia immediately after concussion, apparent disorientation and possible seizure-like activity, and, with sufficiently strong impact, cases of premature death (sometimes within 24 h, under certain conditions) (Katzenberger et al., 2013). Using males of the wild-type Canton-S strain of flies, which are known to engage in aggressive behaviors (Chen et al., 2002), we set out to test: (1) whether male flies subjected to TBI would indeed exhibit the longer-term symptom of elevated aggression and (2) whether ketogenic-like dietary treatment could rescue any aspect of post-TBI symptoms, including elevated aggressive behaviors.

Direct administration of KBs or ketone esters as a replacement for an all-out KD is an emerging area of research. Recently-published studies have shown marked biochemical and behavioral effects of direct dietary supplementation with KBs or KB esters. Feeding human subjects KBs or KB esters results in elevated circulating levels of the KBs beta-hydroxybutyrate and acetoacetate, whether the subjects are in the fasted or fed state (Stubbs et al., 2017). Additionally, KB ester supplementation has resulted in amelioration of neuropathological symptoms in a mouse model (Kashiwaya et al., 2013) and a human case (Newport et al., 2015) of Alzheimer’s disease. KD and KB treatments have also proven efficacious in Drosophila seizure models: a KD is effective against seizures in an ATP61 model of mitochondrial encephalomyopathy (Fogle et al., 2016), and our own group has shown that direct addition of the KB beta-hydroxybutyrate to a standard high-carbohydrate fly diet can reduce seizure-like activity (SLA) in the *eas* (ethanolamine kinase) mutant bang-sensitive seizure strain of *Drosophila* (Li et al., 2017). Based upon these studies, we hypothesized that wild-type Canton-S flies fed a diet supplemented with putatively-neuroprotective KBs would exhibit improvements in post-TBI behavioral abnormalities. Further, we hypothesized that any observed KB effects on behavior might be mediated by K_ATP_ channels, as these channels play a role in other disease models (such as seizure) where a reduction in neuronal excitability could be linked to tempering the severity of neuropathological states (Giménez-Cassina et al., 2012; Li et al., 2017).

## Materials and Methods

### Fly Strains and Husbandry

All flies used for these experiments were males of the Canton-S wild-type *Drosophila* strain. Flies were group-housed on grain- and agar-based food (*see below*) in standard 8.5-cm-long, 2.5-cm-diameter cylindrical polystyrene vials in an incubator set at 25°C, and on a 12-h:12-h light/dark cycle. Following eclosion as adults, flies were separated by sex on the day of concussive banging or fictive banging (*see below*).

### Fly Diets and Pharmacology

All fly diets were based on a standard Bloomington Formulation diet (termed the “control diet”; “Nutri-Fly,” Genesee Scientific, San Diego, CA, United States) with 4.9 mL propionic acid (Fisher Scientific, Hampton, NH, United States) per of liter of food, added as an antifungal agent. Control diet nutrient ratios were approximately 46.7 carbohydrates:7.4 protein:1 fat by mass. Ketone body (KB)-supplemented diets contained 2 mM of the KB sodium *R*-,*S*-beta-hydroxybutyrate (β-HB) added to the control diet. This concentration is consistent with concentrations used in previous *in vitro* and *in vivo* studies (e.g., Ma et al., 2007; Tanner et al., 2011; Li et al., 2017), as well as with those measured in rats on a KD (Appleton and DeVivo, 1974; Jarrett et al., 2008). Drug treatments included the K_ATP_ channel blocker tolbutamide (at a concentration of 200 μM) and the K_ATP_ channel opener diazoxide (at a concentration of 600 μM), both drugs administered either alone (added directly to the control diet food) or in addition to βHB. All chemicals were obtained from Sigma Chemical (St. Louis, MO, United States) unless otherwise noted. For all drug conditions, and most dietary conditions, flies were raised on their given diet from birth (oviposition of embryos) through eclosion from the pupal casing as adults, and were returned to their previous diet after TBI induction (*see* below). Some cohorts of flies had their diets switched from KB-supplemented to control diet (KB-to-CD switch), or vice versa (CD-to-KB switch) after banging on day 5 post eclosion, and remained on the new diet for 3 days until aggression testing. We found that Canton-S flies’ pre-eclosion development took about 10 days on average, resulting in a total of roughly 15 days on each diet before TBI induction (for most experiments) and 18 days before aggression testing.

### Head Trauma Induction

**FIGURE 1:**
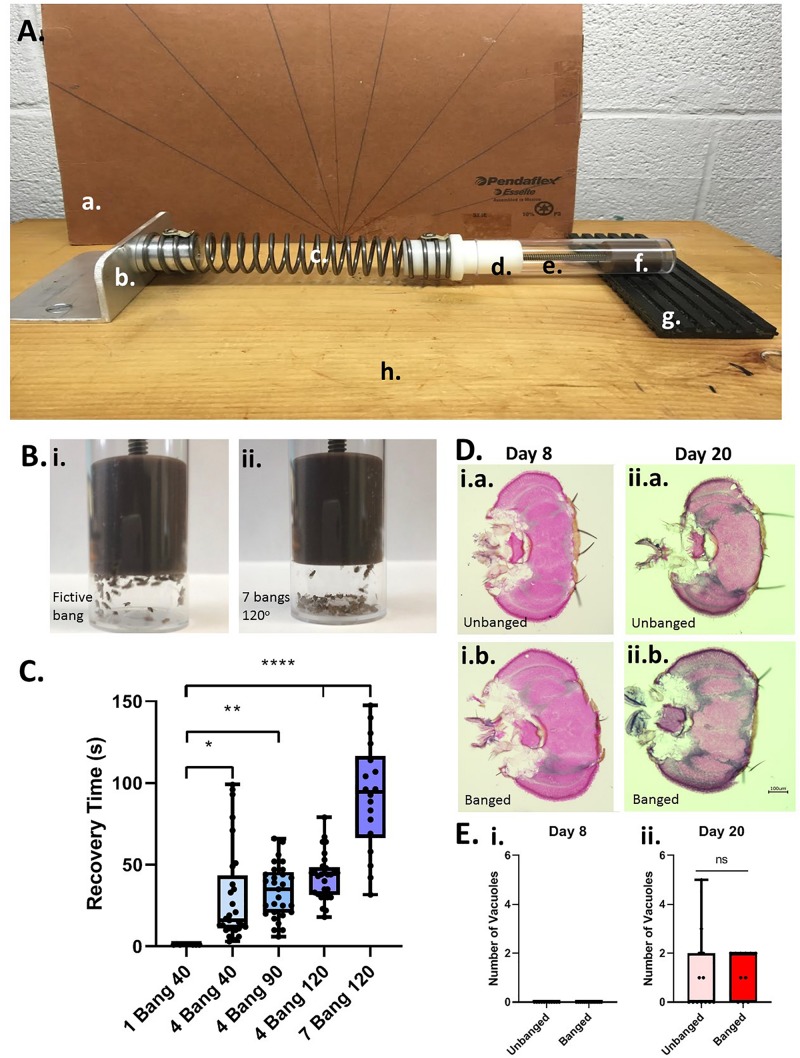
Modeling TBI in *Drosophila* with a modified high-impact trauma (HIT) device. **(A)** Modified HIT device (“banger”) with protractor; based on Katzenberger et al. (2013). The device consists of: (a.) a protractor, (b.) a spring clamp, (c.) a spring, (d.) a vial plug, (e.) a fly vial (bang chamber), (f.) a stopper/plunger to restrict flies to the base of the vial, and (g.) a stiff rubber mat (“TBI pad”), all affixed to (h.) a wooden board. The HIT device was set up in front of the protractor to measure bang angle. **(B)** Fly ataxia and incapacitation post-banging. The majority of flies banged at the highest bang intensity applied (7 bangs at 120 degrees; right panel) exhibited pronounced post-bang ataxia as compared with their unbanged counterparts (left panel). **(C)** Increased number and intensity of bangs increases recovery time from banging. Flies were subjected to increasing bang number and force: 1 bang at 40°, 4 bangs at 40°, 4 bangs at 90°, 4 bangs at 120° and 7 bangs at 120°. For flies fed a standard control diet, there was a steady increase in recovery time from banging with increasing bang intensity. The longest recovery times recorded were for flies subjected to 7 bangs at 120°. A significant difference was detected amongst the distributions of recovery times; *p* < 0.0001, one-way ANOVA. Recovery at higher bang intensities was significantly longer than that for flies banged at 1 bang 40°: ^∗^*p* < 0.05, ^∗∗^*p* < 0.01, ^****^p < 0.0001; Dunn’s multiple comparisons test. Data presented as follows: Center line, median; box boundaries, top of 3rd and bottom of 2nd quartile; whisker ends, upper and lower range of data. Each data point represents the average recovery time for all flies in a given vial. **(D)** Coronal brain sections of flies subjected to the 7 bangs-120° TBI protocol (“banged”) or to fictive banging (“unbanged”) on day 5 post eclosion. Left: Day-8 post-eclosion flies. Right: Day-20 post-eclosion flies. **(E)** Counts of large (>10 μM) vacuolar brain lesions per brain section. For banged and unbanged 20-day post-eclosion flies, no significant difference in the number of such lesions was detected (*p* = 0.90; Student *T* test).

To induce head trauma in flies (as a model for human TBI), we used a variant of the “HIT” device first described in 2013 (Katzenberger et al., 2013). Our HIT device consists of a wooden board, with attached spring clamp, spring, and a vial plug with a plunger/stopper to restrict flies to the base of the vial, a removable fly banging chamber (a vial), and a rubber TBI pad, backed by a hand-made cardboard protractor to measure the angle at which the spring is released (*see* Figure 1A). For all experiments that were followed by long-term behavioral testing of lifespan studies, we placed a number (5–20) of day-5 post-eclosion adult flies, without CO_2_ anesthesia, in an empty cylindrical (2.5 × 8.5 cm) polystyrene fly husbandry vial, and attached the vial to the HIT device using the stopper on the device. We termed one single concussive strike on the HIT device a Big Adverse Neurotrauma-Generating event, or “BANG” event; this acronym (“BANG”) is rendered in lowercase letters throughout the remainder of the text. Flies were banged under 5 different conditions: 1 bang at a 40° angle as measured on the HIT device protractor, 4 bangs at 40°, 4 bangs at 90°, 4 bangs at 120°, and 7 bangs at 120°. Successive bangs were executed within 1–2 s of each other. Control-condition (“unbanged” or “fictively banged”) flies were placed in the HIT device and then removed, with no banging event.

### 24-H Mortality Index (MI-24) Measurements and Lifespan Studies

For short-term mortality studies, we determined the mortality index 24 h after TBI induction (percent of flies dead after 24 h; termed “MI-24”, after Katzenberger et al., 2013). Sets of experimental flies of ages 0–5 days post-eclosion were subjected to the 7 bang-120° TBI protocol. Other sets of experimental flies of only ages 0 and 5 days post-eclosion were subjected to fictive banging. For each condition of age, diet, and banging, approximately half of the vials were transferred to the HIT device with CO_2_ anesthetization and half were transferred without CO_2_ anesthetization five minutes prior to concussive events (banging). Additionally, roughly half of the flies were raised on the control diet till the day of banging; half on KB-supplemented food. For flies banged at 7 bang-120°, between 4 and 15 vials (median 8 vials) containing between 6 and 34 flies per vial (median 23 flies) were used for each condition of age, diet, and anesthesia. For unbanged (fictively banged) flies and flies banged at 4 bang-40°, exactly 8 vials each containing 20 to 27 flies per vial were used for each condition of age, bang, diet, and anesthesia.

After fictive banging or recovery from banging, flies were returned (without anesthesia) to fresh food vials containing the diet on which they were raised. 24 h later, the number of total flies and number of dead flies in each vial was recorded and the percentage of dead flies calculated and reported as the MI-24. Because the use of CO_2_ anesthesia prior to banging appeared to increase mortality (*see* Table 1), we did not use CO_2_ for any other subsequent experiments.

**TABLE 1:** Mortality index 24 h after bang or fictive bang (MI-24).

	***No CO_2_ anesthesia***		***With CO_2_ anesthesia***	
**BANG condition**	**Day 0 flies**	**Day 5 flies**	**Day 0 flies**	**Day 5 flies**
No bang	0.0 ± 0.0%	0.0 ± 0.0%	0.0 ± 0.0%	0.0 ± 0.0%
4 bang-40°	0.0 ± 0.0%	0.0 ± 0.0%	0.0 ± 0.0%	0.0 ± 0.0%
7 bang-120 °	8.16 ± 2.03%**	0.95 ± 0.64%	11.96 ± 2.18%***	6.01 ± 2.28%

*Mortality index 24 h (MI-24) after TBI. Average MI-24 is zero in all cases, except for the 7-bang-120° paradigm; ^∗∗^*p* < 0.01, ^∗∗∗^*p* < 0.001: significantly different from MI-24 values under the same conditions of age and anesthesia. Percentages reported as mean ± SEM.*

For long-term longevity studies, we used four groups of flies—each group numbering 10 males in each of 5 vials—and recorded longevity observations for all four groups simultaneously, in parallel: experiments were initiated on the same date and started from day 0 post-eclosion for all flies. We performed this assay on two separate occasions and pooled the data, resulting in 100 males total per group. Group conditions were: banged, control diet (CD); banged, KB-supplemented diet (KB); unbanged, CD; unbanged, KB. Banged flies were subjected to the 7 bang-120° TBI protocol without CO_2_ anesthesia on day 5 post eclosion, then returned to fresh food vials containing the diet on which they were raised. Number of dead flies in each vial was recorded until all flies were dead.

### Recovery Time From Head Trauma

Immediately following induction of head trauma, most flies typically remained supine and largely motionless at the bottom of the vial. Recovery time for each fly was measured from the moment of the final bang of each condition until righting of the animal with sustained upright posture and resumption of normal locomotion (including negative geotaxis). Measurements of recovery time were made using video recordings of each TBI event by scorers blind to dietary treatment condition. Recovery time was reported as an average time for each vial.

### Histology for Anatomical Scoring

For cryostat preparation, flies were group-housed and separated on day 8 or day 20 post-eclosion (3 days or 15 after day-5 bang or fictive bang). Male flies were selected out and put on dry ice for preservation. Flies were then transferred into a block of OCT (Optimal Cutting Temperature Embedding Medium, Thermo Fisher Scientific, Waltham, MA, United States) and snap frozen to separate the heads. Blocks of OCT containing male heads were transferred to a cryostat (Leica CM3050S, Buffalo Grove, IL, United States) and cut at 25 μm. Slides containing sections were then stained using a standard hematoxylin and eosin (H & E) protocol (*see below*) and mounted. Stained slides were imaged with a 10x objective lens using a Keyence BZ-X700 microscope (Keyence, Itasca, IL, United States) with the Bright-field/Phase Contrast setting. H & E-stained sections were scored and measured by an experimenter blind to treatment condition of the flies. Sections with poor qualitative ratings were excluded from analysis of number of vacuolar lesions greater than 10 μM diameter.

### Hematoxylin and Eosin Staining

Slides with 25 μm sections of male *Drosophila* heads were hydrated in PBS. Sections were then run through a dehydration gradient (25, 50, and 75% ethanol) for 5 min each, followed by 60 dips in 100% ethanol and 95% ethanol. After a brief rinse with tap water, slides rested in the hematoxylin solution for 5 min and were washed briefly. Next, slides were dipped into acid alcohol 6 times, washed, and rested in lithium carbonate for 2 min. Slides were placed into a tap water bath for 5 min, dipped in 80% ethanol 30 times, and placed in eosin phyloxine-B solution for 90 s. Next, slides were dehydrated in 95% ethanol for 30 dips followed by 100% ethanol for 90 dips. Lastly, slides were dipped 30 times into xylene followed by a one minute bath in xylene. Slides were mounted with toluene and allowed to dry before imaging.

### Wing-Damage Assay

Males group-housed with females and fed a control diet were either banged on day 5 post eclosion or subjected to fictive banging (unbanged), then returned to fresh control-diet vials. On day 8 post eclosion (3 days after TBI or fictive TBI), animals were anesthetized with CO_2_ and had their wings clipped off with iridectomy scissors. The wings were mounted on glass coverslips and imaged using light microscopy. Wings damaged by the wing-harvesting process were discarded. Scorers blind to treatment condition rated the wings on a 1-8 damage scale following an established rating system (Davis et al., 2018).

### Behavioral Aggression Measurements

Following recovery after induction of concussive trauma in the HIT device, flies were returned to a fresh food vial in their incubator until day 8 post eclosion (day 3 post-banging), when they were subjected to an aggression assay. All flies were returned to the same diets on which they had previously been reared, except the flies subjected to a dietary switch (*see above*). For each aggression assay, two male flies were placed—via buccal aspirator, without CO_2_ anesthesia—in an enclosed 1.5 cm square behavioral arena, and given several minutes to acclimate to the chamber. After 2–3 min, a decapitated female was introduced into the chamber to induce male-male aggression. Activity was recorded on an iPhone6 camera (Apple Inc., Cupertino, CA, United States) for 180 s from placement of the female into the chamber. Aggressive behaviors were scored on two different dimensions: frequency of aggressive events, which included the recording of four aggressive behaviors: fencing (hitting opponents with forelegs), rearing/lunging, charging at an opponent, and wing flaring while facing an opponent; and latency to the first recorded aggressive event of any of the four types. Latency was measured as the time between placement of the female in the chamber and the first identified unambiguous aggressive event. Scorers were blind to the prior treatment condition of the flies being tested, recorded, and scored.

### Negative Geotaxis Assays

For negative geotaxis assays, 10 flies were placed in a closed cylindrical chamber made of two empty husbandry vials taped together at their open ends and inscribed with height markings in units of 1-cm increments. Flies were gently tapped to the bottom of the upright chamber, at which point their progress in climbing upwards against gravity (negative geotaxis) was recorded on an iPhone8 camera (Apple Inc., Cupertino, CA, United States) under dim red light to avoid the confound of positive phototaxis. Two parameters were recorded from the moment of down-tapping (denoted time zero): average percentage of flies to reach 6 cm height climbed after 10 s, and the average height climbed after 30 s. Flies were banged at 7 bangs-120° on day 5 post-eclosion and tested for motility on day 8 post-eclosion.

### Cold Exposure Trauma and Electric Shock Trauma

For cold exposure trauma, 25–30 flies (in empty, plugged fly husbandry vials) were placed, horizontally, into a plastic bucket containing dry ice for 10 s, then removed onto the room-temperature benchtop for 10 s, and this cold-warm cycle was repeated six times.

For electric shock, 30–35 flies were placed in a custom-built shock tube (Con-Elektronik, Greussenheim, Germany) controlled by an Arduino processor (Adafruit Industries, New York, NY, United States) linked to a desktop computer (Dell, Round Rock, TX, United States). Flies were shocked at 120 V, with a shock duration of 1.5 s, repeated every 5 s, over the course of one minute, for a total of 12 shock pulses. Flies subjected to cold exposure and electric shock on day 5 post-eclosion were returned to their 25°C incubator on a standard control diet and subsequently tested on day 8 in the aggression assay (*see above*). Flies were only ever subjected to one type of trauma and were all raised on the control diet.

### Statistics and Data Presentation

Statistical analysis was performed using Microsoft Excel (Microsoft; Redmond, WA, United States) and Prism (GraphPad; La Jolla, CA, United States) software. Proportion data (percentages and fixed-scale scores) were first unconstrained by logit transformation; for the purpose of avoiding undefined values, data points of zero were assigned values of 0.000001. Statistical comparisons on the transformed data were made using the Student *T*-test for single pairwise comparisons. Statistical comparison of differences between recovery times or logit-transformed MI-24 values on identical dietary conditions used the Dunn’s multiple comparisons test. Pairwise Mann–Whitney *U* tests were used to compare effects on aggression of dietary treatments within the same banging paradigm. Analysis for single pairwise comparisons between unbounded data sets also used the Student *T*-test.

For count data (e.g., number of aggressive events) the Kruskal–Wallis test was used to detect the existence of differences amongst distributions, followed by pairwise Mann–Whitney *U* tests between distributions, with the Bonferroni correction for multiple comparisons (*see below*).

The D’Agostino and Pearson test was used to determine the normality of the distribution of data points for continuous data sets (e.g., recovery times, latencies to first aggressive event). An analysis of variance (ANOVA) was then performed to detect differences amongst distributions: one-way ANOVA for comparisons with a single factor (e.g., bang intensity, different diet treatments) followed either by Dunn’s multiple comparisons test (recovery) or by a *post hoc* Tukey’s multiple comparisons test (latencies); one-way ANOVA not assuming equal standard deviations (i.e., the Brown–Forsythe and Welch ANOVA), followed by a *post hoc* Games-Howell’s multiple comparisons test, with individual variance computed for each comparison (trauma and drug treatments); two-way ANOVA for comparisons with two factors (e.g., diet vs. bang treatments), followed by Fisher’s least significant differences test (latencies in different diet and bang treatments).

Unless otherwise noted, data are presented as follows: Center line, median; box boundaries, top of 3rd and bottom of 2nd quartile; whisker ends, upper and lower range of data.

For lifespan studies, survival curves were plotted as Kaplan-Meier plots, which were compared using the Mantel-Cox log-rank test with a Bonferroni correction for six pairwise comparisons. A table of *p*-values adjusted for the Bonferroni correction for six pairwise comparisons follows:

**Table:** 

Significance level	*p* < 0.05	*p* < 0.01	*p* < 0.001	*p* < 0.0001
Adjusted for *k* = 6	*p* < 0.0083	*p* < 0.00167	*p* < 0.000167	*p* < 0.0000167
Significance markers	^∗^	^∗∗^	^∗∗∗^	^****^
				

## Results

### High-Impact Trauma Induces Markers of TBI-Like Head Trauma in Young-Adult Male *Drosophila*, Including Slowed Recovery and Elevated Mortality Post-banging

To model TBI in flies, we developed a modified HIT device—a fly “banger” —(*see* Figure 1A) similar to that used in recent publications (Katzenberger et al., 2013, 2015) to deliver head-trauma-inducing bangs (“big adverse neurotrauma-generating events”) to young-adult males (days 0 to 5 post-eclosion) of the wild-type Canton-S *Drosophila melanogaster* strain. Making use of our fly banger, we banged Canton-S flies fed on a standard high-carbohydrate control diet at several different combinations of forces and frequencies (*see* Materials and Methods and Figure 1). As reported previously (Katzenberger et al., 2013), we observed that following the traumatic hit (the “bang”), flies exhibit an immediate loss of activity—lying supine against the floor-side wall of their bang vial (sometimes experiencing tremors and motor convulsions)—and a delay in both self-righting and a return to normative locomotion, including negative geotaxis (Figure 1B). We interpreted these visible signs of abnormal locomotor behavior as being indicative of head trauma.

We termed the duration of incapacitation “recovery time:” the time elapsed between the last bang and a resumption of normative movement. For flies banged at day 5 post-eclosion, we found that, with an increase in severity of banging (greater angle of banging and/or number of bangs), came an increase in recovery time (Figure 1C). We chose the longest-recovery (and highest-intensity) banging paradigm (7 bangs at an angle of 120 degrees; 7 bangs-120°) for the remainder of most of our experiments, to mimic severe head trauma as the result of multiple concussions, such as a contact-sport athlete might experience in a live contest. We measured an average force of roughly 10 Newtons (9.92 ± 0.12 N; average of 21 bangs) for 7 bangs-120°, and a fly-and-food-containing vial mass of around 20 grams (18.54 ± 0.13 grams; average of 3 vials), from which we calculated an average acceleration of around 500 m/s^2^, or roughly 50 times the acceleration due to gravity (50 *g*). This number is in line with accelerations at contact (ranging from around 10 *g* to over 80 *g*) measured in college football players during live, helmeted practices (Mihalik et al., 2007).

We wished to confirm whether concussive banging induces sufficient trauma to produce measureable neuroanatomical lesions and post-bang mortality, as has been previously described (Katzenberger et al., 2013).

For a quantitative anatomical measure of neurological damage, we counted large vacuolar lesions measuring greater than 10 μM diameter in the brains of flies sectioned either 3 or 15 days after bangs or a fictive bang delivered on day 5 post-eclosion (thus, flies aged day 8 or day 20 post-ecolsion, respectively). Contrary to previous reports (Katzenberger et al., 2013), we detected no such lesions in sections from the brains of flies three days after bang or fictive bang (Figures 1D,E, left-hand panels). However, we did measure one or more large vacuolar lesions at this size threshold in some of the brains of flies sectioned 15 days after banging (in 9/14 sections from different animals; 64%) or after fictive bang (in 6/13 sections from different animals; 46%); however, there was no significant difference in number of vacuoles between banged and unbanged conditions for these 20-day-old flies (Figures 1D,E, right-hand panels).

We then assessed a more-readily-measureable marker of presumptive head trauma: elevated levels of post-trauma mortality [reported as “MI-24”: the mortality index 24 h after banging (Katzenberger et al., 2013)]. As a baseline point of comparison, we found (as to be expected) absolutely no death in flies belonging to the unbanged control group, at any age or condition tested (0% MI-24 in all cases; *see* Table 1). We also found absolutely no death in any flies banged at the 4 bang-40° paradigm. However, we did detect non-trivial MI-24 levels at the 7 bang-120° bang intensity: for flies banged on day 0 post-eclosion, without CO_2_ anesthesia, average MI-24 reached over 8% (*see* Table 1). Nonetheless, in contrast to previously published results reporting high post-TBI mortality—at levels of 25% or more (Katzenberger et al., 2013)—under our protocols, the resultant MI-24 levels were considerably lower, even under our highest-intensity banging paradigm (7 bang-120°). Indeed, for 0-day-old male adult flies not subjected to CO_2_ anesthesia prior to banging, we found at most 18.2% MI-24 in one individual vial. For 5-day-old male adult flies, we observed almost no mortality at all: only 0.95 ± 0.64% (mean ± SEM; 2 out of 163 flies total in multiple separate, parallel, identically treated vials) of control-dieted flies banged on our protocols at 7 bangs-120° died within 24 h of banging.

This discrepancy may be partly explained by the lack of anesthetic use in our initial experiments, as compared with a previously reported use of CO_2_ anesthesia (Katzenberger et al., 2013, 2015). To test this idea, we subjected a cohort of flies to prior CO_2_ anesthesia before banging. Under the 7 bang-120° condition, we did find that MI-24 was non-zero in flies anesthetized with CO_2_ prior to banging, for flies banged both at day 0 and at day 5—and significantly greater for day 0 post-eclosion flies (*see* Table 1)—although, again, these MI-24 levels were not as high as previously reported (Katzenberger et al., 2013).

Overall, while not in all cases as pronounced as indicated by previous reports (Katzenberger et al., 2013, 2015), our measurements of recovery time and MI-24 subsequent to the highest-intensity banging paradigm under our protocols did suggest the induction of head trauma analogous to TBI. We therefore proceeded to further examine other behavioral outcomes of high-intensity TBI-inducing bangs; to best position ourselves to be able to perform longer-term behavioral and longevity studies, we chose to bang at 7 bangs-120°, without CO_2_ anesthesia, in flies aged to day 5 post-eclosion, as they exhibited low levels of post-bang mortality, while still showing detectable signs of head trauma.

### Banged Flies Exhibit Elevated Male–Male Aggressive Behaviors

**FIGURE 2:**
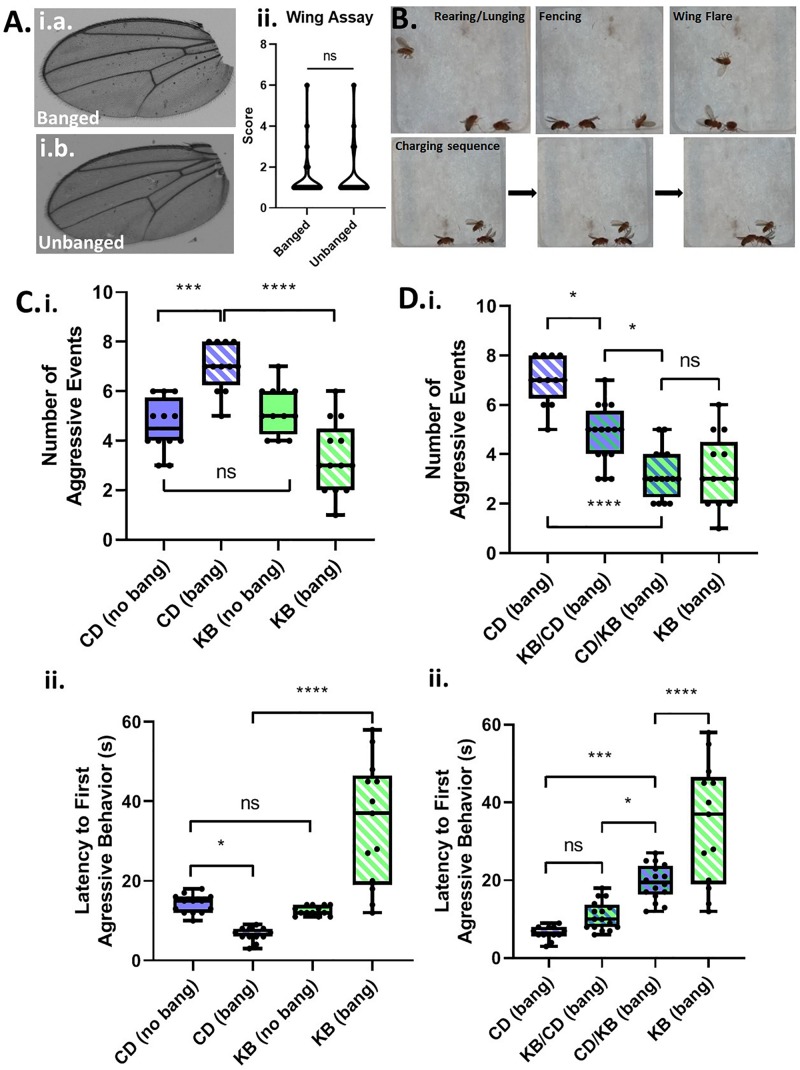
Concussive strikes increase aggressive behavior; this effect is prevented by ketone bodies. **(A)** No increase in post-TBI aggressive behavior is detectable by wing-damage assays. A median wing-damage score of 1 was recorded for group-housed male flies, whether or not subjected to banging. No significant difference was detected between damage scores; *p* = 0.9720, Student *T*-test following logit transform of raw data. All flies in this and subsequent figures were subjected to the 7 bang-120° paradigm. **(B)** Aggressive behavior is exhibited by pairs of male flies previously subjected to TBI. Pairs of males were acclimated to an enclosed behavioral chamber prior to introduction of a decapitated female to induce aggression. *Top*: Males engaging rearing/lunging, fencing, and wing flaring. *Bottom*: A sequence of still images captured from a video recording showing a charging event. **(C)** TBI elevates male-male aggression; ketone body supplementation reduces post-TBI aggression. (i) Male flies on either a control diet or a KB-supplemented diet were subjected to an aggression-behavior assay 3 days after either TBI induction or fictive banging performed on day 5 post-eclosion. Over 180 s of recorded encounter, banged flies on the control diet (CD) exhibited a significantly higher median number (7.0 ± 1.0) of aggressive events as compared with unbanged counterparts (4.5 ± 0.5 aggressive events). For unbanged flies on a KB-supplemented diet, median aggressive event number was 5.0 ± 1.0, which is comparable to that of unbanged control-fed flies. For banged flies on the KB-supplemented diet, median aggressive event number was significantly lower than that of control-dieted banged counterparts, at 3.0 ± 1.0. Data reported as median ± median absolute deviation (MAD). For number of aggressive events, a significant difference (*p* < 0.0001, Kruskal–Wallis test) was detected amongst the distributions of counts; significant differences were detected between counts of aggressive events for banged control-dieted flies as compared with both unbanged CD-fed and banged-fed KB flies; ^∗∗∗^*p* < 0.001, ^****^p < 0.0001 pairwise Mann–Whitney *U* test, with Bonferroni correction for 6 pairwise comparisons. (ii) Banged control-diet-fed flies exhibited a significantly shorter latency to first aggressive event (6.50 ± 0.50 s; mean ± SEM) than that of unbanged flies on the control diet (14.15 ± 0.65 s). Notably, for the distribution of latencies of banged CD-fed flies, each data point was lower than all of those in the distribution of latencies for unbanged CD-fed flies. There was no significant difference between unbanged KB-fed flies’ latency to aggression (12.54 ± 0.33 s) as compared with that of unbanged CD-fed flies. Banged KB-fed flies exhibited a mean latency to onset of aggression of 34.38 ± 4.33 s, which was significantly longer than that of their banged CD-fed counterparts. For latency to first aggressive event, a significant difference (*p* < 0.0001, two-way ANOVA) was detected amongst the distributions of latencies; significant differences were detected between distributions of latencies to first aggressive event for banged KB-dieted flies, as compared with all other conditions; ^∗^*p* < 0.05, ^****^p < 0.0001, Fisher’s least significant differences test; no direct correction for multiple comparisons. **(D)** A switch from control to KB-supplemented diet on the day of TBI also reduces aggression. (i) Male flies switched to the KB diet for 3 days after banging (CD-to-KB switch; “CD/KB”) exhibited a low median number of aggressive events, comparable to that for banged flies fed a KB diet their whole lives. For number of aggressive events, a significant difference was detected amongst the distributions of counts (*p* < 0.0001, Kruskal–Wallis test). Number of aggressive events were, for: control-diet only, 7.0 ± 1.0; KB-supplemented-to CD diet, 5.0 ± 1.0; CD-to-KB, 3.0 ± 1.0; KB-supplemented-only, 3.0 ± 1.0. Data reported as median ± median absolute deviation (MAD). ^∗^*p* < 0.05, ^****^p < 0.0001; significantly different from the indicated dietary treatment by Dunn’s multiple comparisons test. Data for number of aggressive events for CD banged and KB banged conditions identical to that from (**C**, i). (ii) Male flies switched to the KB diet for 3 days after banging (CD-to-KB switch; “CD/KB”) exhibited a longer latency to first aggressive event than flies fed a control diet their whole lives or given the KB-to-CD switch (“KB/CD”). For latency to first aggressive event, a significant difference was detected amongst the distributions of latencies by a one-way ANOVA (*p* < 0.0001). Latencies to first aggressive event were, for: control-diet only, 6.50 ± 0.50 s; KB-supplemented-to CD diet, 10.88 ± 0.92 s; CD-to-KB, 19.50 ± 1.14 s; KB-supplemented-only, 34.38 ± 4.33 s. Data reported as mean ± SEM. ^∗^*p* < 0.05, ^∗∗∗^*p* < 0.001, ^****^p < 0.0001; significantly different from indicated dietary treatment by *post hoc* Tukey’s multiple comparisons test with single-pooled variance. Data for latency to first aggressive events for CD banged and KB banged conditions identical to that from (**C**, ii).

A common symptom of TBI-induced CTE in humans is personality change, including explosivity and increased aggressive behavior (McKee et al., 2012). By analogy, we reasoned that male Canton-S flies, which are known to exhibit aggression (Chen et al., 2002) as part of their suite of normal behaviors, would exhibit elevated aggressive behaviors when subjected to head trauma. We group housed males following either fictive banging or actual TBI induction on day 5 post eclosion, and examined wings for damage—an anatomical proxy for aggressive behavior—three days later on day 8. However, we could detect no appreciable damage at all in the wings of banged flies as compared with those that were not banged (Figure 2A), indicating that we could not use this morphological marker for analysis of aggression.

Although we were unable to record evidence of elevated aggression by the wing-damage assay, we reasoned that if TBI does elevate aggression, we could detect such a change by analysis of active aggressive behaviors in live pairs of male flies. For our assays, we videographically recorded male-male aggression in a closed chamber (with a female body present as a trigger to induce aggression over mating access; *see* Figure 2B) and later manually analyzed the data offline with scorers blind to treatment condition. We compared aggression in banged and unbanged flies fed a standard (control) high-carbohydrate diet for their whole lives, and found that banged flies exhibited significantly elevated aggression, as measured both by a higher number of aggressive events and a shorter latency to first aggressive event (*see* Figure 2C and Tables 2A,B). Although the decrease in latency to first aggressive event exhibited only moderate statistical significance, in making a comparison of banged and unbanged flies, we emphasize nonetheless that every recorded latency for banged control-diet-fed flies was lower than every latency for their unbanged counterparts—there was zero overlap between the distributions—showing that banged flies were quicker to engage in triggered aggression. These results pointed to a marked effect of concussive head trauma on increasing aggression in male flies.

**TABLE 2A:** Statistical outcomes (*p*-values) for counts of numbers of aggressive events.

	**Banged, KB**	**Unbanged, CD**	**Unbanged, KB**
Banged, CD	0.000002 ^****^	0.000037 ^∗∗∗^	0.00054 ^∗∗^
Banged, KB	–	0.026, *ns*	0.0013 ^∗∗^
Unbanged, CD	–	–	0.18, *ns*

Following Kruskal–Wallis test (*p* < 0.0001; significant differences amongst means), pairwise Mann–Whitney *U* tests, with Bonferroni correction for 6 pairwise comparisons was used to compare conditions. Uncorrected *p*-values are reported, with asterisks denoting significance with Bonferroni correction. ^∗∗^*p* < 0.01; ^∗∗∗^*p* < 0.001; ^****^p < 0.0001.

**TABLE 2B:** Statistical outcomes (*p*-values) for measurements of latency to first aggressive event.

	**Banged, KB**	**Unbanged, CD**	**Unbanged, KB**
Banged, CD	<<0.0001 ^****^	0.018 ^∗^	0.058, *ns*
Banged, KB	–	<<0.0001 ^****^	<<0.0001 ^****^
Unbanged, CD	–	–	0.606, *ns*

Following two-way ANOVA (*p* < 0.0001; significant differences amongst means), Fisher’s Least Significant Difference Test was used to compare means between conditions; Fisher’s LSD does not account for multiple comparisons. ^∗^*p* < 0.05; ^****^p < 0.0001.

### Administration of a KB-Supplemented Diet Reduces Aggression in Banged Flies

One mechanism by which head trauma is proposed to cause brain damage is excitotoxic neuronal death (Blaylock and Maroon, 2011); this elevated cell death may in part help explain the induction and development of dysfunctional behavioral outputs reported in cases of TBI-induced CTE. Thus, we hypothesized that supplementation of a normal control diet with putatively-neuroprotective KBs could serve to reduce the elevation of aggressive behavior observed in banged files. We therefore treated different cohorts of flies on four dietary regimens: (1) a standard control diet (CD; as above) or (2) a KB-supplemented diet (KB), both diets for the duration of the experiment, from oviposition of embryos through aggression testing; (3) a KB diet through the day of banging, followed by a switch to control food for three days until aggression testing (KB-to-control switch); and (4) a control diet through the day of banging, followed by a switch to KB-supplemented food for three days until aggression testing (control-to-KB switch).

Between control-only and KB-only dietary treatments, we observed no significant differences in aggressive behavior in flies that were not banged: unbanged flies on the KB-supplemented diet exhibited a median number of aggressive events and average latency to first aggressive event comparable to those of flies on the control diet. However, quite strikingly (and in line with our hypothesis), as compared with the banged CD-fed cohort, the banged cohorts of Canton-S flies fed on KB-supplemented food their entire lives exhibited a pronounced and statistically significant reduction in aggressive behaviors on day 3 post-TBI (day 8 post-eclosion). This marked reduction in aggressive behavior in banged flies raised on the KB dietary treatment compared with banged flies raised on the control dietary treatment was apparent both in a lower number of aggressive events and a longer latency to first aggressive event (Figure 2C). Indeed, every latency to first aggressive event recorded for banged KB-fed flies was greater than all latencies recorded for banged CD-fed flies.

To test the effects on post-TBI aggression of duration and regimen of previous exposure to the KB supplemented diet, we assayed aggression in banged flies fed on both a KB-to-control switch diet and on a control-to-KB switch diet. Surprisingly, although they were KB-dieted for only the three days between banging and testing, banged flies on the control-to-KB switch exhibited a marked and highly significant reduction in aggression, as compared with CD-fed banged counterparts (Figure 2D). Indeed, for measures of number of aggressive events in banged CD-to-KB fed flies, the reduction was comparable to that of flies fed a KB diet their whole lives. Additionally, we found that flies treated with the KB-to-control switch also exhibited a slightly, but significantly reduced number of aggressive events, as compared with banged control-diet-fed counterparts. These KB-to-CD-fed flies also appeared to exhibit a slightly greater median latency to first aggressive event, in line with a general trend observed for reduced aggression with peri-bang administration of KBs. However, this moderate increase in latency for the KB-to-CD switch, unlike that for the CD-to-KB switch, was not significantly different from the latency measured in control-diet-fed flies. Taken together, these results suggest that post-concussive treatment with KBs is more efficacious than pre-treatment.

It is of particular note that, between control-only and KB-only dietary treatments, we observed no significant differences in aggressive behavior between groups of differently dieted unbanged flies (Figure 2C and Tables 2A,B). Additionally, for the comparison of number of aggressive events, we also detected no significant difference in the distributions of counts between CD-fed unbanged flies and KB-fed banged flies (Table 2A). Overall, these results suggest that the KB dietary intervention can positively influence post-TBI pathological behavior, but will not adversely impact normative behavior in animals that have not been subjected to head trauma.

### Changes in Aggressive Behavior Are Specific to Concussive Head Trauma and Are Not the Result of General Motility Deficits

We reasoned that the observed changes in apparent aggression might occur as a result of motility changes (especially motility deficits in cases of lower measured aggression). Additionally, we speculated that elevations in aggressive behavior following TBI might be attributable to generalized neuronal death or neural-circuitry alterations that might result from exposure to any kind of trauma, and not necessarily head trauma specifically.

**FIGURE 3:**
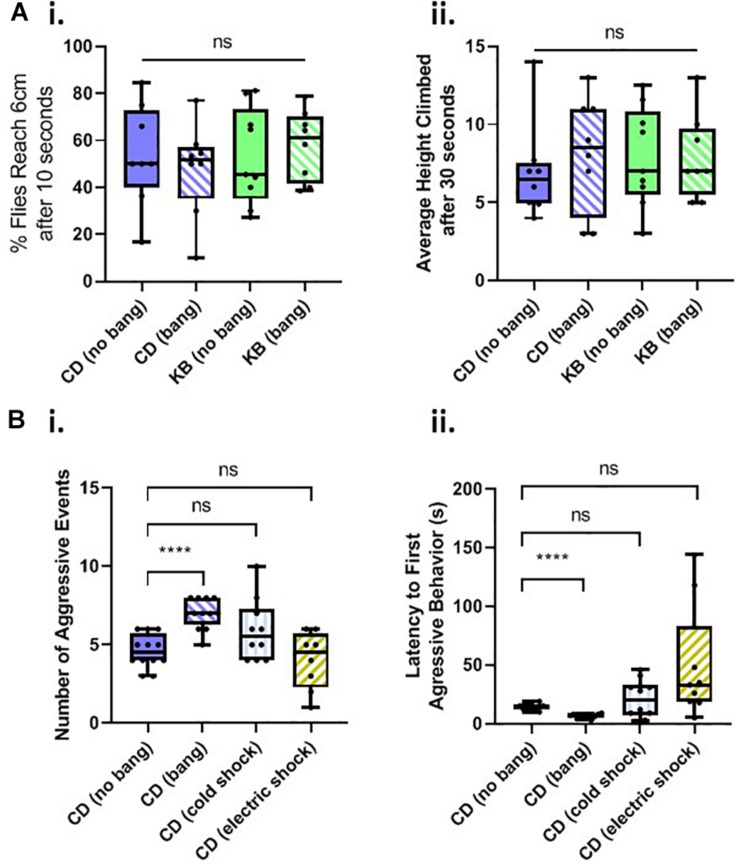
Changes in aggressive behavior are not caused by motility deficits and are specific to head trauma. **(A)** Neither head trauma nor diet condition affects basal motility in a negative geotaxis assay. (i) For measurements of the percentages of flies reaching 6 cm after 10 s, no significant difference was detected amongst the distributions of percentages across bang and dietary conditions (*p* = 0.8830 Kruskal–Wallis test). (ii) For measurements of flies’ average height climbed after 30 s, no significant difference was detected amongst the distributions of heights, across bang and dietary conditions; overall interaction, *p* = 0.5935; dietary condition, *p* = 0.6090; bang condition, *p* = 0.7551, two-way ANOVA. **(B)** Non-concussive traumas of cold exposure and electric shock do not increase aggressive behavior. (i) For measurements of the number of aggressive events, a significant difference was detected amongst the conditions (*p* < 0.001, Kruskal–Wallis test). No significant difference was detected between counts of aggressive events for comparisons of unbanged flies to both cold-exposed and electrically-shocked flies (pairwise Mann–Whitney *U* test.) There was a statistically significant difference between counts of events for unbanged and banged flies (^****^p < 0.0001, without Bonferroni correction), as reported in Figure 2C.i. (ii) For measurements of latencies to the first aggressive event, a significant difference was detected amongst the conditions; *p* < 0.001, one-way ANOVA not assuming equal standard deviations (Brown–Forsythe and Welch ANOVA). No significant difference was detected between latencies to first aggressive event for comparisons of unbanged flies to both cold-exposed and electrically shocked flies (Games-Howell’s multiple comparisons test). All animals for trauma experiments were fed the control diet for their whole lives. Data for number of, and latency to first, aggressive events for CD-fed banged and unbanged conditions identical to that from Figure 2C.

A common, robust, and repeatable fly behavior is to spontaneously climb up the walls of their vials in the opposite direction of gravity (*see*, e.g., Ali et al., 2011), a behavior termed negative geotaxis. We therefore tested flies on a negative-geotaxis assay of climbing ability (*see* Materials and Methods) to determine whether basal motility is affected by head trauma, diet, or both. We fed cohorts of flies on two diets: a control diet (CD) or a KB-supplemented diet, for their whole lives. As in the aggression experiments, we either banged or fictively banged flies on day 5 post-eclosion, and tested climbing ability on day 8. We measured both average percentage of flies to reach 6 cm height climbed after 10 s, and the average height climbed after 30 s for all flies. We found that neither diet nor bang condition induced any significant difference in motility, as measured by these two components of the negative-geotaxis assay (Figure 3A). These results led us to conclude that any changes in behavior observed in the aggression assay were specific to alterations in motivated aggressive behavior itself and not to motility deficits or increases.

We then tested whether other trauma types besides head trauma would induce changes in aggressive behavior. We subjected flies fed a control diet for their whole lives to cold-exposure trauma or to electric-shock trauma on day 5 post-eclosion, and tested their behavior in an aggression assay 3 days later, on day 8 post-eclosion (Figure 3B). We found that neither the number of, nor the latency to, aggressive events in Canton-S males exposed to extreme cold or electric shock differed significantly from those measures of aggression recorded for control-dieted males subjected to no head trauma at all (For electric shock, a few outlier male-male pairs did exhibit a rather long latency to first aggressive event although this result would be indicative of reduced, not elevated post-traumatic aggression: the animals were slower, not quicker, to engage in bouts of aggressive behavior.) These results therefore led us to conclude that the increase in aggressive behavior observed in banged control-dieted flies was specific to head trauma induced by the HIT device.

### The Effects of the Ketone Body β-HB on Post-TBI Aggressive Behaviors Appear to Be Partially Mediated by K_ATP_ Channels

There exists an established link between the activity of ATP-sensitive potassium (K_ATP_) channels activity and KB effects in brain slice models (Ma et al., 2007; Tanner et al., 2011). A role has also been demonstrated for K_ATP_ channels in ameliorating seizure-like activity in *in vivo* mouse (Giménez-Cassina et al., 2012) and fly (Li et al., 2017) models. We therefore chose to investigate whether K_ATP_ channel activity might also mediate the observed KB effects in this *Drosophila* model of TBI using the K_ATP_ blocking drug tolbutamide and the K_ATP_ opener diazoxide.

**FIGURE 4:**
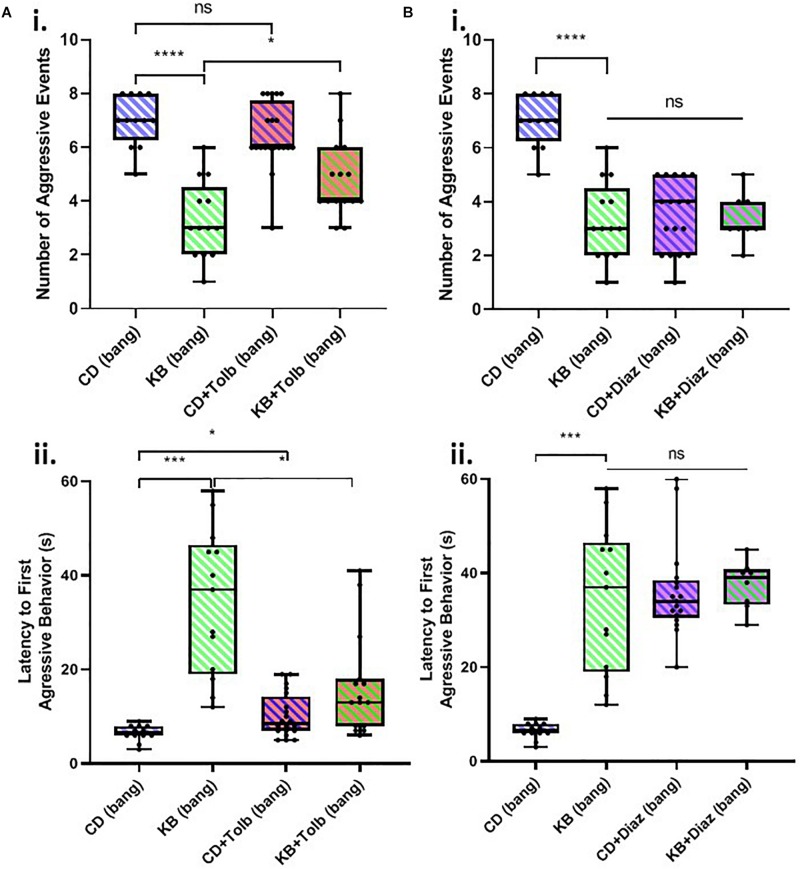
K_ATP_-channel-targeting drugs affect post-TBI aggression. **(A)** The K_ATP_-channel-blocking drug tolbutamide (200 μM) added alone to control food exerts little effect on aggression in banged flies, but may partially block the ketone body effect on reducing post-TBI aggression. Tolbutamide alone added to the control diet mildly lengthened latency to first aggressive event (^∗^*p* < 0.05) but did not significantly affect the number of aggressive events. Tolbutamide added on top of the KB-supplemented diet exerted a slight but significant effect via a reduction in aggression latency (^∗^*p* < 0.05), as compared with values recorded for KB-supplement-dieted flies. **(B)** The K_ATP_-channel-opening drug diazoxide (600 μM) mimics on its own, and does not augment, the ketone body effect on reducing post-TBI aggression. Both diazoxide on its own, and diazoxide added on top of the KB-supplemented diet, resulted in a large, significant reduction in number of aggressive events, and an increase in aggression latency, as compared with these measures for banged flies fed a control diet. (All comparisons ^****^p < 0.0001, except for comparison of latency to first aggressive event between CD and KB bang treatments; ^∗∗∗^*p* < 0.001.) No significant difference was detected amongst any of the diazoxide or KB conditions, neither for number of, nor latency to first, aggressive event. Data for number of, and latency to first, aggressive events for CD banged and KB banged conditions identical to that from Figure 2C. All flies banged at 7 bangs-120°.

We found that tolbutamide alone added to the control diet had no statistically-significant effect on the number of aggressive events in post-TBI (banged) male-male pairs and resulted in only a small (but nonetheless significant) increase in latency to the first aggressive event in banged flies (Figure 4A). In examining the impact of tolbutamide on the established KB effect of reducing post-TBI aggression, we found that the addition of tolbutamide on top of the KB-supplemented diet appeared to partially abrogate KB effects on post-TBI aggression. Tolbutamide treatment of KB-fed flies resulted in a slightly, but significantly greater number of, and shorter latency to first, aggressive events following banging, as compared with the values observed in banged flies on the KB-supplemented diet alone.

In contrast, the application of diazoxide alone to the control diet resulted in measures of banged flies’ post-TBI aggression that were dramatically reduced (both lower number and longer latency) as compared with those measured in banged control-dieted flies (Figure 4B). These measures of post-TBI aggression were not significantly different from those recorded in banged flies fed on the KB-supplemented diet. Moreover, the addition of diazoxide on top of KB-supplemented diets resulted in no further significant reduction in aggressive behaviors in flies subjected to head trauma.

These results showed that blocking K_ATP_ channels with tolbutamide appeared to partially reverse the KB effect on reducing one measure of post-bang aggression, and that opening K_ATP_s with diazoxide mimicked the KB effects. Taken together, this evidence suggests that the above-reported KB effects on post-TBI aggression are, at least in part, mediated by K_ATP_ channels.

### KB Treatment Dramatically Extends Lifespan for Flies Not Subjected to High-Impact Trauma

**FIGURE 5:**
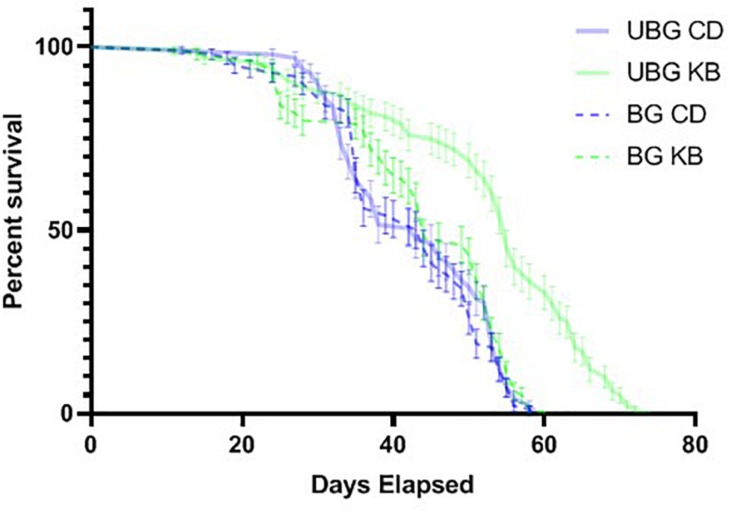
Ketone bodies extend lifespan in unbanged flies. Flies were either banged at the highest bang intensity (7 bangs at 120°; BG), or subjected to fictive TBI induction (“unbanged”; UBG). Separate vials of flies were then monitored daily for animal death over a roughly 11-week period after banging or fictive banging, until all animals had died. Kaplan–Meier plots of post-banging lifespan reveal a pronounced longer lifespan (*p* < 0.0001, Mantel-Cox log-rank test) for unbanged flies fed a KB-supplemented diet, as compared with the lifespans of flies for all other conditions of diet and banging. Day 0 is the first day of post-eclosion adult life. Flies were banged or fictively banged on day 5. CD, control diet; KB, KB-supplemented diet. Individual data points plotted as mean ± SEM for percent survival across 10 independent vials in each condition.

Our observed KB effects on aggression, coupled with previously reported effects of TBI on reducing lifespan in flies (Katzenberger et al., 2013), and our own results pointing to an effect of KBs in reducing MI-24 in banged flies first anesthetized with CO_2_ (Table 3), piqued curiosity regarding whether KBs might exert an effect on post-TBI longevity in flies subjected to our highest-intensity banging paradigm. Therefore, we tracked lifespan following TBI (7 bangs-120°) or fictive banging (in the unbanged controls), for both dietary conditions (CD and KB; flies fed on the given diet from the egg stage through death). We observed relatively long lifespans in all conditions: over 85% survival at 30 days post-eclosion for all groups; median lifespans all over 40 days (Figure 5 and Tables 4A,B), regardless of treatment. While control-dieted flies subjected to TBI had the shortest average lifespan (41.7 ± 1.1 days; mean ± SEM), the banged CD-fed flies’ lifespan did not differ significantly from that for their unbanged counterparts (42.8 ± 1.0 days). Thus, again in contrast with previous reports (Katzenberger et al., 2013), our results showed that banging does not induce high levels of mortality, at least under our specific protocols.

**TABLE 3:** Mortality index 24 h (MI-24) after TBI for CD- and KB-fed flies.

	***No CO_2_ anesthesia***		***With CO_2_ anesthesia***	
**Diet condition**	**CD**	**KB**	**CD**	**KB**
Day 0 flies	8.16 ± 2.03%	3.68 ± 1.77%	11.96 ± 2.18%	3.04 ± 1.05% ^∗^
Day 5 flies	0.95 ± 0.64%	0.85 ± 0.85%	6.01 ± 2.28%	0.59 ± 0.59% ^∗^

*Ketone bodies moderately reduce MI-24 only in day 0 and day 5 males subjected to CO_2_ anesthesia; ^∗^*p* < 0.05, Mann–Whitney *U* test. Percentages reported as mean ± SEM; data for CD-fed flies reproduced from Table 1.*

**TABLE 4A:** Unbanged, KB-supplemented flies exhibit the longest lifespan.

**Condition**	**Mean lifespan (days ± SEM)**
Control diet (CD), Banged	41.7 ± 1.1
Control diet, Unbanged	42.8 ± 1.0
KB supplement diet (KB), Banged	43.4 ± 1.2
KB supplement diet, Unbanged	52.2 ± 1.4

*Banged flies on the control diet had the shortest average lifespan and unbanged flies on the KB diet had the longest average lifespan across any of the conditions.*

**TABLE 4B:** Raw numerical outcomes (*p*-values) for statistical comparisons of lifespan measurements.

	**Banged, KB**	**Unbanged, CD**	**Unbanged, KB**
Banged, CD	0.0699	0.6159	<0.0001^∗∗∗^
Banged, KB	–	0.3336	<0.0001^∗∗∗^
Unbanged, CD	–	–	<0.0001^∗∗∗^

*Lifespans were compared using the Mantel-Cox log-rank test with the Bonferroni correction for 6 total pairwise comparisons between the 4 different dietary and banging conditions. ^∗∗∗^*p* < 0.001 (corresponding to *p* < 0.00017, following Bonferroni correction.).*

Of note, we did find that KB supplementation dramatically extended lifespan in unbanged flies—by over 8 days on average (to 52.2 ± 1.4 days) as compared with lifespans not only for their unbanged control-fed counterparts but also with lifespans for their banged KB-fed and banged CD-fed counterparts. However, we found no effect of KBs on extending lifespan in banged flies: although mean lifespan for banged flies fed a KB-supplemented diet was slightly longer (43.4 ± 1.2 days) than that for banged flies on the CD, there was no statistically significant difference in lifespan between the two banged conditions (Figure 5 and Tables 4A,B). Thus, we observe that that KBs may extend lifespan in unbanged flies—in agreement with the finding that KBs extend lifespan in *C. elegans* (Edwards et al., 2014) (for a review of possible mechanisms, *see* Veech et al., 2017)—but not in banged flies.

## Discussion

In a modified *Drosophila* model of concussive head trauma, using young-adult males of the wild-type Canton-S strain, we have recapitulated aspects of previously-reported results (Katzenberger et al., 2013) showing that concussive banging results in the manifestation of several markers of TBI-like damage, including slowed recovery from banging (following ataxia and incapacitation), and elevated post-bang mortality within 24 h (MI-24). In our hands, progressively higher-intensity banging resulted in progressively longer post-bang recovery times, and an increase (from 0%) in mortality measured as MI-24. Our modified TBI methodology nonetheless results in less post-trauma mortality than previously reported (Katzenberger et al., 2013), thus permitting robust post-TBI behavioral analyses.

As a consequence, we were able to develop a model for the study of post-TBI aggression. This model produced the clear demonstration that flies subjected to high-intensity banging exhibit elevated post-TBI male-male aggressive behaviors, in line with symptoms reported for human contact-sport athletes with TBI-induced CTE (McKee et al., 2012). Additionally, and strikingly, we have demonstrated the marked efficacy of a simple dietary intervention—KBs—to ameliorate this specific post-TBI pathological behavior of elevated aggression. As a dietary supplement, direct administration to fly food of the KD metabolite beta-hydroxybutyrate (a KB), either prior to and following TBI induction (“banging”), or for only the 3 days immediately after banging, resulted in dramatic and significant effects both on reducing the frequency, and delaying the time to onset, of aggression in post-TBI young-adult male flies.

This ketone-body effect on post-TBI aggressive behavior is unlikely to be the result of a mere overall reduction in general neural activity, nor of a targeted reduction in motor-specific activity, for at least three reasons. First, for the high-intensity banging (7 bangs at 120°) to which animals used for all aggression assays were subjected, prior KB administration exerted no appreciable effect on average rates of recovery from TBI. Animals on both control and KB diets exhibited similar average times required for a return to regular locomotion (including negative geotaxis) from ataxia and disorientation immediately subsequent to high-impact (7 bangs-120°) trauma (Table 5). Additionally, negative-geotaxis tests for basal motility performed 3 days after banging were comparable across all treatments of flies: KB and control-diet treated, banged and unbanged. These results indicate the absence of the induction of any specific immediate or long-term motor deficit in KB-treated flies. Second, with regards to aggressive behavior, KB-dieted flies behaved similarly to flies on a normal control diet: there was no statistically significant difference between latency to, or frequency of, aggressive events in KB-treated animals as compared with CD-fed counterparts when the animals were not subjected to the TBI protocol. This result also suggests that KB treatment itself has no effect on basal activity in the absence of head trauma. Taken together, these results strongly support the conclusion that there is no generalized depression or alteration of motor activity resulting from KB treatment, TBI induction, or both. This absence of KB effects on basal motility and aggression is all the more notable in that it suggests that KB treatment could conceivably be applied as an ameliorative intervention for post-TBI symptoms without deleterious effects on at least some aspects of normative behavior and activity. Finally, the observed effects of banging on aggressive behaviors appear to be specific to head trauma, since subjecting flies to the traumas of extreme-cold exposure and electric shock resulted in no statistically significant increase in aggression, as compared with non-traumatized controls, by all measures. These results suggest that the observed post-TBI behavioral change of elevated aggression arises from dysregulation in aggression-related neural circuitry emerging as a direct and specific consequence of concussive banging, and is not a general outcome of putative neural damage resulting from any arbitrary trauma exposure.

**TABLE 5:** KB treatment improves recovery time at two lower bang intensities; it does not significantly improve recovery time at 7 bangs-120°.

	**Median recovery time**	
**Bang condition**	**Control diet (CD)**	**KB supplemented diet**
1 bang, 40°	1.0 ± 0.0	1.0 ± 0.0
4 bangs, 40°	16.0 ± 10.0^∗^	12.5 ± 5.5 #
4 bangs, 90°	35.0 ± 12.0^∗∗^	22.5 ± 11.5 ^∗∗∗^
4 bangs, 120°	44.0 ± 9.0^****^	23.0 ± 10.0^∗∗∗^ ##
7 bangs, 120°	94.5 ± 22.5^****^	77.1 ± 21.9^****^

For comparisons with recovery times at 1 bang 40^o^ within the same dietary treatment: ^∗^*p* < 0.05, ^∗∗^*p* < 0.01, ^∗∗∗^*p* < 0.001, ^****^p < 0.0001; Dunn’s multiple comparisons test. For comparisons with recovery times between dietary treatments at the same banging paradigm: *#p* < 0.05, *##p* < 0.001, pairwise Mann–Whitney *U* tests. Numerical data for CD reflects that plotted in Figure 1C; for all data, values reported as median ± MAD.

Previous studies have shown evidence that neural glycolysis is reduced in the brains of rodents immediately following TBI (Yoshino et al., 1991; Thomas et al., 2000). This decrement in metabolism may make neuronal survival post-injury more challenging, as it can serve to lower cellular energy charge. It is therefore possible that one cellular-level mechanism by which ketone body application may rescue behavioral changes is by counteracting post-TBI metabolic deficits through augmentation of mitochondrial metabolism, even in the face of reduced neuronal glycolysis, thus helping supply energy to stave off cell death in the relevant neural circuits.

At the molecular level, our pharmacological results using drugs targeting the ATP-sensitive potassium (K_ATP_) channel suggest that the effects on post-TBI aggression of the KB β-HB may be at least partially mediated by K_ATP_ channel activity (Figure 4). We found that co-application of the K_ATP_ blocker tolbutamide partially abrogated some of the KB effects on amelioration of elevated post-TBI aggression. At the same time, application of tolbutamide alone to the control diet (CD) maintained counts of aggressive events at levels comparable to those observed on the CD. This result is in line with the idea that neural K_ATP_ channels are thought to remain largely in the closed (inactive) state under normative conditions (*see*, e.g., Tanner et al., 2011): tolbutamide on its own would therefore be expected to have few, if any, pronounced effects on neuronal excitability, and thus neurally mediated behavior, at baseline. More dramatically, diazoxide—a K_ATP_ opener—by itself mimicked KB effects on aggression, while addition of diazoxide on top of KB-supplemented food showed no additional effect on reducing aggression. This latter result thus strongly suggests, through experimental occlusion, that KBs and diazoxide are operating through a similar mechanism, which is highly likely to be K_ATP_ channel activation.

K_ATP_ channels have previously been implicated as mediators of metabolic effects on reducing neuronal firing rate in brain-slice models (Ma et al., 2007), and seizure activity in whole-animal models (Giménez-Cassina et al., 2012; Li et al., 2017). K_ATP_ channels are known to exhibit an increased open probability in the presence of KBs (Tanner et al., 2011). Here, we add to the body of evidence implicating the K_ATP_ channel as one molecular mediator of KB effects, and in a disease model other than seizures. If, indeed, excitotoxicity plays an important role in the pathogenesis of post-TBI CTE, as has been suggested (Blaylock and Maroon, 2011), membrane hyperpolarization through K_ATP_ activation by KBs—which is known to slow action potential firing in central neurons (Ma et al., 2007)—would represent a viable mechanism by which such over-excitation in neural circuits could be tempered.

It bears noting that these pronounced effects on reducing post-TBI aggression were achieved by mere administration of the KB beta-hydroxybutyrate alone, not a full KD, and that the KB effects on aggression were apparent and rather strong even by treating banged flies on a KB-supplemented diet for just 3 days following TBI. Several studies in adult rats have shown positive effects of a full KD regimen following brain injury. A few studies in rodent models (Hu et al., 2009a, b) have reported positive beneficial effects of 3 days of post-TBI KD (in this case, a completely carbohydrate-free KD) on reducing cell death and expression of apoptotic markers, as well as mitochondrial damage and oxidative stress. A KD has also been found to reduce both apoptosis and contusion size in young-adult rats following controlled cortical injury (CCI) (Prins et al., 2005). Additionally, evidence has been found for positive effects of a week or more of post-injury KD on improvement of motor deficits and memory (measured by the Morris water maze test) in young-adult rats after CCI (Appelberg et al., 2009). In addition, a relatively recent study in *Drosophila* found beneficial effects of post-injury starvation—a water-only diet, even for just a few hours, which would be predicted to induce at least moderate ketosis—on mortality following TBI (Katzenberger et al., 2016).

However, to our knowledge, ours is the first animal model to date that has used direct application of KBs to a standard high-carbohydrate diet (as opposed to a full KD, which necessitates low carbohydrate levels) to ameliorate a post-TBI cognitive-behavioral abnormality with KD-like treatments, and the first such model using *Drosophila*.

Identification of effective treatments for TBI is of increasing importance. Annually, U.S. hospital emergency rooms (ERs) receive over 2 million visits for TBI-related events, accounting in 2013 for nearly 2% of all ER visits (Taylor et al., 2017). TBI-related ER visits have increased over the last decade, notably, with recent rates of TBI in males roughly 18% higher than that in females (Taylor et al., 2017). Our results, in conjunction with those of other groups, support the idea of metabolic interventions—notably caloric restriction (CR), or treatments that mimic some aspects of CR, such as the KD (Wilder, 1921)—as a potentially effective mode of post-TBI therapy. However, while a full KD is very restrictive in terms of permitted amounts and proportions of nutrient intake (with accompanying difficulty in administration and compliance), and fasting can be challenging even for short (12 h or less) periods of time, supplementation of a normal diet with KBs is straightforward and simple, thus indicating such treatment as a possible therapeutic intervention for TBI.

In addition, our results demonstrate that a lifelong adherence to a KD or a KB-supplemented diet is not necessary for the observed KB effects on reduction in post-TBI aggression. Following TBI induction, a simple dietary switch, from a lifelong high-carbohydrate diet (like that of many Americans and other Westerners; *see* for example, Cordain et al., 2005) to the same high-carbohydrate diet supplemented with KBs, resulted in a marked decrease in the number of aggressive events (on par with the number for flies fed a KB-supplemented diet their whole lives), as well as a large increase in the latency to the first aggressive event. Similar effects (though not nearly so dramatic) were found in cases where flies were dieted on the KB-supplemented diet for their whole lives, then shifted to a control diet after concussion. We thus note, first, that KB administration only following TBI (and not for an entire previous lifespan) may be sufficient to incur neuroprotective effects; and second, that some of the effects of KBs lingered even after the dietary supplement was removed, in line with observations made about the persistence of post-weaning effects of KDs in epileptic juveniles (Martinez et al., 2007).

Taken together, our experiments establish a functional model for the study of post-TBI elevations of aggression and strongly suggest that KD-like dietary treatments following TBI may help contribute to amelioration of deleterious post-traumatic effects on behavior and neural health. Further research is warranted to determine first, whether this mode of treatment may be efficacious for other molecular and cellular indicators (e.g., cell death, tauopathy) or cognitive-behavioral symptoms (e.g., learning and memory deficits) of TBI and/or CTE; and second, to elucidate further the molecular signaling pathways and neural circuits mediating any such dietary-metabolic effects.

## Data Availability Statement

The datasets generated for this study are available on request to the corresponding author.

## Author Contributions

DL, KV, SB, PM, and GT conceived the experiments. DL, KV, SB, MF, JLF, JRF, JCF, FL, SM, PM, TO’T, and MR conducted the experiments and scored animal behavior. DL, KV, JD, and GT performed data analysis. DL, KV, SB, JD, TO’T, and GT wrote the manuscript.

## Conflict of Interest

The authors declare that the research was conducted in the absence of any commercial or financial relationships that could be construed as a potential conflict of interest.
